# Relationship between food addiction and the use of appetite-reducing drugs among medical students

**DOI:** 10.1192/j.eurpsy.2026.11374

**Published:** 2026-07-31

**Authors:** T. C. Garcia, C. Garcia, M. Campos, T. Hortencio

**Affiliations:** Faculty São Leopoldo Mandic, Campinas, Brazil

## Abstract

**Introduction:**

Medical students are exposed to high academic pressure, emotional stress, and intense cognitive demands, conditions that may contribute to dysfunctional eating patterns, including food addiction. This condition is associated with dysregulation of the dopaminergic reward system and may lead to strategies to control appetite and weight, such as the use of appetite-reducing medications, often without adequate indication or monitoring.

**Objectives:**

To estimate the prevalence of food addiction among medical students and investigate whether the use of appetite-reducing drugs is associated with this condition.

**Methods:**

A cross-sectional study was conducted with medical students from a college in the state of São Paulo, Brazil. The modified Yale Food Addiction Scale 2.0 (mYFAS 2.0) and a structured questionnaire on the use of appetite-reducing drugs were applied. After ethics approval, data were analyzed using descriptive statistics and association tests (Chi-square and Fisher’s exact test) to examine relationships between food addiction, sex, and medication use.

**Results:**

A total of 546 students participated; 70.9% were women and 89.7% were aged 18–25 years. Most reported being white (89.9%) and enrolled in their first undergraduate degree (88.1%). Appetite-reducing drug use was reported by 30.2%, mainly lisdexamfetamine (11%), semaglutide (7.5%), and methylphenidate (7.1%). Food addiction was identified in 10.6% of students, with 5.3% classified as severe. Women were 2.29 times more likely to present food addiction (OR = 2.29; 95% CI = 1.09–4.81; p = 0.028). Students who used appetite-reducing drugs had a higher likelihood of food addiction (OR = 2.88; p < 0.05).

**Conclusions:**

There was a high prevalence of appetite-reducing drug use among medical students, and this group showed a significantly higher rate of food addiction, suggesting the use of pharmacological strategies to control eating behavior. Food addiction was more frequent among women. These findings reinforce the need for targeted nutritional education, psychological support, and monitoring of appetite-reducing medication use among medical students to encourage healthier coping strategies and prevent disordered eating behaviors.

**Disclosure of Interest:**

None Declared

